# Trends in North American Newspaper Reporting of Brain Injury in Ice Hockey

**DOI:** 10.1371/journal.pone.0061865

**Published:** 2013-04-17

**Authors:** Michael D. Cusimano, Bhanu Sharma, David W. Lawrence, Gabriela Ilie, Sarah Silverberg, Rochelle Jones

**Affiliations:** Division of Neurosurgery, Department of Surgery, Injury Prevention Research Office, Keenan Research Center, St. Michael's Hospital, University of Toronto, Toronto, Ontario, Canada; University of Victoria, Canada

## Abstract

The frequency and potential long-term effects of sport-related traumatic brain injuries (TBI) make it a major public health concern. The culture within contact sports, such as ice hockey, encourages aggression that puts youth at risk of TBI such as concussion. Newspaper reports play an important role in conveying and shaping the culture around health-related behaviors. We qualitatively studied reports about sport-related TBI in four major North American newspapers over the last quarter-century. We used the grounded-theory approach to identify major themes and then did a content analysis to compare the frequency of key themes between 1998–2000 and 2009–2011. The major themes were: perceptions of brain injury, aggression, equipment, rules and regulations, and youth hockey. Across the full study period, newspaper articles from Canada and America portrayed violence and aggression that leads to TBI both as integral to hockey and as an unavoidable risk associated with playing the game. They also condemned violence in ice hockey, criticized the administrative response to TBI, and recognized the significance of TBI. In Canada, aggression was reported more often recently and there was a distinctive shift in portraying protective equipment as a solution to TBI in earlier years to a potential contributing factor to TBI later in the study period. American newspapers gave a greater attention to ‘perception of risks’ and the role of protective equipment, and discussed TBI in a broader context in the recent time period. Newspapers from both countries showed similar recent trends in regards to a need for rule changes to curb youth sport-related TBI. This study provides a rich description of the reporting around TBI in contact sport. Understanding this reporting is important for evaluating whether the dangers of sport-related TBI are being appropriately communicated by the media.

## Introduction

Concussions and other forms of mild traumatic brain injuries occur at least 1.7 million times a year in North America and account for about 75% of all traumatic brain injuries (TBI) [1], [2], [3]. Sport-related head trauma is a common cause of TBI in youth, and every year in North America, nearly half million youth aged 14 years or less need hospital-based care for this injury [3], [4]. The Centers for Disease Control and Prevention (CDC) recently declared that sport concussions are a “silent epidemic” and that they deserve further research [3].

Repeated concussions and TBI are of particular concern as they may cause life-lasting cognitive and psychosocial deficits [5], [6]. These injuries are common in all contact sports, but those who play ice hockey are at particular injury risk [7], [8], [9], [10]. The potential long-lasting effects of TBI suggest that these injuries are an important threat to public health [11]. Prevention of sport-related TBIs requires multifaceted approaches that consider issues related to the nature of play and the culture existent within ice hockey [12], [13].

At elite levels, such as the National Hockey League (NHL), aggression (i.e., a purposeful physical act driven by intent to cause physiological or psychological harm) is valued and has been considered to be an effective success strategy [14], [15]. Aggressive players are quickly recognized for their style of play by coaches, management, other players, and fans [16]. Moreover there still exists, among the sports community, a widespread attitude that concussions are “a part of the game” and resiliency to medical council is considered a sign of “toughness” [17]. These issues hinder prevention and treatment efforts and call for research to address these concerns.

An attitude that stresses “toughness” and “ruggedness” of players who can “heroically brush off” injuries often pressures players to neglect their own safety and health for the game [16]. Social learning theory proposes that such aggressive play is encouraged and fostered in ice hockey culture, and by learning of the positive rewards of aggression in ice hockey, aggressive behaviour continues within the sport [18]. Since aggressive play in ice hockey can increase injury incidence by making high-speed collisions more likely and by fostering an “intent-to-harm” attitude among players [19], understanding the media portrayal of TBI in ice hockey is important for evaluating whether the clinical severity of these injuries is being appropriately communicated.

To better understand how the mass media and popular culture report TBI in sports like ice hockey, we studied a sample of newspaper articles. The manner by which newspapers portray ice hockey-related TBIs and how this has changed over time has not yet been examined. The purpose of our paper was to inductively identify themes in Canadian and American newspaper reports of ice hockey-related TBIs, and to determine if, over time, there has been any change in the content and nature of these reports. Our goal was to understand the reporting of these injuries and the implications of this reporting.

## Methods

### Sample

We performed a qualitative analysis of newspaper articles published, between 1985 and 2011, in the: Chicago Tribune (CT), New York Times (NYT), Toronto Star (TS), and Vancouver Sun (VS). We selected these newspapers based on the size of their readership. Furthermore, we sought to represent: (1) Canada and the United States: (2) east and west coast ice hockey media reports; and (3) both original-six and expansion-era ice hockey teams. We chose newspapers with a local nature rather than a national coverage because they would likely report in more detail about local hockey market issues. Furthermore, by analyzing local instead of national newspapers, it was possible to evaluate whether the ice hockey culture in different localities was consistent within and across localities. The four newspapers had a cumulative average weekly circulation of over 13 million copies in 2011 [20], [21].

### Data collection

We retrieved all newspaper articles electronically from the ProQuest™ database, an online periodical index part of the Cambridge Information Group. To locate and retrieve newspaper articles that discussed ice hockey brain injuries we used the following search terms: Hockey (in citation and document text) AND “concuss*” OR “head injur*” OR “head trauma” OR “brain injur*” OR “brain trauma” OR “banged head” OR “bell rung” OR “conscious*” (in citation and abstract). Key scientific and colloquial terms were included in the search algorithm as newspapers were likely to use both sets of terms. We included newspaper articles in our study if they referred to one or more of the key terms a minimum of three times within the body and/or headline of that article.

Newspaper articles published before January 1^st^, 1985 were not available from ProQuest™; we collected data after this point until the end of the 2011 NHL season (June 15^th^, 2011). We reviewed and analyzed articles from each of 4 time intervals, that is1985–1989, 1990–1999, 2000–2009, and 2010–2011. The number of analyzed articles published in each time interval varied. A total of 541 newspaper articles were analyzed; 49, 185, 187, 120 were published in 1985–1989, 1990–1999, 2000–2009, and 2010–2011, respectively. Furthermore, the number of newspaper articles published by each print media source varied, as 120, 126, 140, 155 articles were analyzed from the CT, NYT, TS, and VS, respectively.

### Data analysis

We used the grounded-theory approach as a framework for a thematic analysis to provide rich descriptions of the nature and character of newspaper articles on the topic of interest over time [22]; categories and themes were allowed to emerge from the data inductively and were not pre-identified by *a priori* hypotheses [23], [24]. We recorded data into meaningful pieces of information known as meaning units (MU) [25]. After we coded MUs, they were used to create broad categories, which were later classified into specific themes and sub-themes. We used the constant comparison method throughout the coding process in that as each MU emerged, it was compared to other MUs that were similarly categorized to determine appropriate classification. In addition, we did a content analysis by counting the frequency with which MUs occurred within the sample text [23].

All retrieved newspaper articles were imported as text files into HyperRESEARCH™ qualitative research software, formerly used in qualitative healthcare investigations [26], [27], [28]. The MUs from each article were coded using HyperRESEARCH™. Three members of the research team, who met on a weekly basis to discuss the coding process, independently completed all coding. We continued to review newspaper articles until we reached saturation of information in each time interval. In all cases, analyzing 50 articles per newspaper per time interval exceeded data saturation [29].

To investigate specific trends over time, we focused on the time periods 1998–2000 and 2009–2011. Since the time period of 2009–2011 is four years after the 2004–2005 NHL lockout, we chose a comparison time period that was four years before this lockout (i.e. 1998–2000). We compared these time periods by means of a thematic and content analysis to explore the nature of and frequency distribution of MUs between time periods and geographical regions (i.e., Canada and America). Three members of the team reviewed the articles, and collaboratively discussed any discrepancies in the classifications of MUs. We assessed coder agreement by means of inter-rater reliability (Cronbach's alpha for coding of MUs was 0.91).

## Results

### Thematic analyses

Five main themes, each comprised of several subthemes, emerged and they are described in **Table 1**. These five themes were highly recurrent across all media sources in all time intervals and were prominent in both countries.

**Table 1:** The five major themes – and associated sub-themes – identified while conducting the analysis.

Theme	Subtheme	Discussion points
Aggression	Aggression as cause of injury	How aggressive play contributes to head injuries
		How many head-injuries could be avoided if aggressive play was minimized
	Contributors to aggression	What fuels aggressive behaviour in players
		Demand for aggressive play
	Attitudes on aggression	The need to curb aggression
		The importance of aggressive play to the culture of hockey
Perceptions of brain injury	Risk perceptions	Perceived clinical severity of injury
		Perceived dangers of repeat injury
		Long-term consequences of brain-injury
	Impact of injury	Impact of injury on team standings and success
		Impact of injury on player short-and long-term health
Equipment	Attitudes on the role of equipment	Equipment as a means to prevent brain injuries
		Equipment as a contributor to brain injuries
	Effectiveness of equipment	The need for better equipment to prevent more brain injuries
		The inability of helmets to prevent brain injuries
Rules & regulations	Attitudes on rules and regulations	The need for more stringent rules and regulations in ice hockey
		The need for more liberal rules and regulations in ice hockey to ‘let the players play’
		The (in)ability of rules and regulations to prevent brain injuries in ice hockey
Youth hockey	Attitudes on youth hockey	The importance of keeping young ice hockey players safe
		The dangers of a brain injury to the development of youth
		The pressure youth face from parents and coaches to return to play as soon as possible

Examples of the discussion points that comprise a given sub-theme are provided above. All Meaning Unit codes (N = 1535) were sorted under one of the above themes.

#### Canadian newspapers

Canadian newspapers disapproved of and condemned needless aggression and violence in ice hockey as expressed in these excerpts:

*I know of no other way of expressing our shame and dissatisfaction with the violence and unnecessary fighting that seems to be the present image for hockey* – VS, 1999

*Canadians were just treated to some amazing hockey at the Olympics and nowhere was fighting or head-hunting seen. The game can survive and thrive without it* – TS, 2010

Canadian newspapers consistently reported that TBIs in ice hockey were serious injuries. However, these newspapers also reported that these injuries are just a part of the game, and that they are essentially an unavoidable occupational hazard.

In the earlier time period, protective equipment was often described as a solution to ice hockey's brain injury problem; wearing more protective equipment was thought to improve player safety. In contrast, during the recent time period, the emphasis shifted distinctly and it was reported that protective equipment makes players feel invulnerable to injury, and this causes them to take more potentially harmful physical liberties.

Canadian newspapers consistently reported how poor the NHL was at enforcing rules on player safety. Although the need for a culture change in ice hockey was consistently discussed, recent reports placed responsibility on the NHL to make the game safer:

*It would be nice to say the NHL is attacking this [concussion] problem with zest, but, as is generally the case with Bettman's aimless administration, the words aren't being backed up with action* – TS, 2000

*To their great discredit, the league and its general managers have dragged their feet on the [brain injury] issue… the league took some half-hearted steps to protect players against the worst effects of head-injuries* – TS, 2011

Recently, reports began to state there is enough inherent excitement in ice hockey given the finesse of the sport that the need for aggressive and violent play is *unnecessary*. **Table 2** provides quotes that illustrate the themes seen in Canadian newspapers.

**Table 2:** Quotes from Canadian newspapers that represent trends in reporting of brain injury in ice hockey.

Theme	1998–2000	2009–2011
Aggression	Gratuitous violence should be removed from the NHL	
	*Without question, the gratuitous violence, hacking, slashing, cross checking and fisticuffs that so dominates the NHL game today is the main factor in my own loss of interest in the NHL* – VS, 1999	*Hockey is such a fast moving and exciting sport. There should be no time for fights, which are increasing arranged in advance by goons who get little ice time otherwise* – VS, 2009
Perceptions of brain injury	Brain injuries are viewed as occupational hazards	
	*It's a physical game, so we all have to accept that there are going to be injuries* – VS, 1999	*As long as people are playing collision sports and are moving at a high rate of speed, there will be some concussions* – VS, 2010
	The need for a culture change with respect to brain injuries	
	*Hockey players from all levels who have suffered concussions and several prominent doctors, neurosurgeons, trainers and officials all agreed that a major shift has to take place in the culture of hockey to deal with the growing problem of brain injuries* – TS, 1998	*What's unacceptable is the willful inability to grasp this issue of serious brain injury in hockey -and every “concussion” is serious, the effects are cumulative, some of the guys now on injury lists are going to suffer early dementias and cognitive loss later in life* – VS, 2011
Equipment	Equipment and brain injuries	
	*Maybe I [player] won't have to recover from (a concussion) again. The precautions I've taken with a new helmet, mouthguard and visor should, hopefully, prevent that* – TS, 1999	*There is talk of upgrading the quality of NHL helmets to reduce head injuries, but New Jersey Devils GM Lou Lamoriello says improved equipment only makes players take more liberties* – VS, 2009
Rules & regulations	Poor governance in the NHL	
	*There is more of an emphasis on supplementary discipline for players guilty of delivering wanton blows to an opponent's head, but those penalties aren't particularly onerous given the damage sometimes done to the victim* – TS, 1999	*First and most obvious, the performance of the league's referees in punishing headshots has been worse than abysmal. Again and again, the refs have failed to step up and administer the most immediate and effective punishment* – VS, 2010
Youth hockey	Severity of youth brain injuries	
	*Concussions - long the most overlooked of serious sports injuries - are especially dangerous for young people because they affect the brain's ability to absorb new information* – TS, 1999	*Various research has shown bodychecking is the leading cause of serious injuries -including concussions and fractures -in kids' hockey* – VS, 2011

#### American newspapers

Similar themes and trends emerged in the American newspaper articles. In both time periods, American newspapers reported that aggression was an integral ingredient to ice hockey and that brain injuries should be accepted as a part of ice hockey. However, both American and Canadian newspapers expressed much more concern recently about the potential long-term clinical impacts of brain injuries. The focus during the earlier time period was to report TBIs only when they occurred to star players, with little mention to the overall extent of the issue:

*The league is concerned about the growing number of concussions that sidelined such headliners as Paul Kariya, Eric Lindros and Pat LaFontaine last season* – CT, 1998

In contrast, the more recent trend has been to describe the broader impact of TBIs to the cross section of the league and to set star players' injuries into the bigger context of the overall problem:

*Repeated concussions can have long-lasting effects and head injuries have caused several hockey players in recent years to end their careers prematurely, including Eric Lindros, Pat Lafontaine and Keith Primeau* – CT, 2009

The serious nature of these injuries was noted in both time periods but the more recent reports stressed the need for a serious culture change within the sport *because* of TBIs:

*Only in the past few years has the NHL dropped its historically cavalier attitude toward concussions* – NYT, 1998

*Calls are proliferating for changes to the culture of a sport that many see as too accepting of reckless body contact and serious injury* – NYT, 2010

Reference to better equipment as a hazard as opposed to a protective factor was also noted in more recent American reports, as well as the need for the league to strengthen rules in an attempt to make the game safer by preventing brain injury. Although the risks of TBI to youth hockey players were consistently reported over time, more recently, calls for the elimination of such injuries to youth became more prominent. **Table 3** provides illustrative quotes from American newspapers.

**Table 3:** Quotes from American newspapers that represent trends in reporting of brain injury in ice hockey.

Theme	1998–2000	2009–2011
Aggression	Aggression as an integral component to ice hockey	
	*It's also my [NHL VP and director of hockey operations] responsibility to keep hockey a physical game. After all, hits happen* – CT, 1999	*Hitting is a big part of the game, and we have to be careful we don't go too far and make the game just a finesse game, just a skating game* – NYT, 2010
	*When it's your turn to take the field, the court or the ice rink, you're ready to hand out a little punishment of your own… you're determined to do so, because that is how, according to today's broadcast sports discourse, you demonstrate athletic superiority* – CT, 1999	*The NHL remains bound by an ethos of toughness, an arena where fighting is tolerated and even encouraged as rough justice, and where playing through concussions and gruesome lacerations are marks of courage* – NYT, 2011
Perceptions of brain injury	Brain injuries are viewed as occupational hazards	
	*By the nature of its violent game, the NHL also can't prevent concussions. They're going to occur every so often* – NYT, 1999	*Hockey is probably the fastest team sport out there, and it can get pretty violent… Getting hurt happens a lot. Nobody is really 100 percent healthy out there* – CT, 2010
Equipment	Equipment and brain injuries	
	*LaFontaine tirelessly advocated ways to prevent serious concussions, stressing the importance of wearing mouth guards and more protective helmets*– NYT, 1998	*Other sports have spent the last several years realizing that safety equipment can bring dangers of its own. Checking in professional hockey became considerably more vicious with the adoption of helmets in the 1970s and '80s* – NYT, 2011
Rules & regulations	The need for stricter rules	
	*The league intends to apply a much stricter standard of supplementary discipline for any deliberate action by a player that is either directed to the head of an opponent or results in an injury to the head* – NYT, 1998	*Under pressure from medical researchers, owners and even players, the general managers are expected to strengthen Rule 48, the league's bylaw governing checks to the head, which was instituted this season* – NYT, 2010
Youth hockey	Severity of youth brain injuries	
	*Two or more significant blows to the head while playing sports can harm teenagers' thinking abilities for years to come* – CT, 1999	*This approach toward eliminating head contact, both incidental and intentional, is critically important for our youth players* – NYT, 2011

### Content analyses

A summary of the number of MUs associated with each theme and newspaper in both time periods is provided in **Table 4**. The findings from newspapers in each country were similar; there were only international, not intra-national, differences in media reporting of ice hockey head-injuries.

**Table 4:** The number of MUs (N = 1535) pertaining to the identified themes, as per time period and geographic region.

Country	Time period	Aggression	Perceptions of brain injury	Equipment	Rules & regulations	Youth hockey
Canada	1998–2000	76	182	11	12	6
	2009–2011	157	122	7	46	33
America	1998–2000	83	247	34	27	18
	2009–2011	97	214	26	88	49

Kruskal-Wallis tests revealed *ns* effects of country, or time period, on each of the identified categories.

Canadian print media discussed aggression in ice hockey equally during both time periods. The content of discussions of TBI in the more recent time period more often dealt with the severity and impact of the injury. Discussion of rules and regulations recently increased in Canada.

In comparison to Canadian newspapers, American newspapers less frequently discussed aggression as a contributor to TBI in ice hockey but more often discussed perception of risks of brain injury. In contrast to Canadian newspapers, American ones more often report on equipment and rules during both time periods. Both American and Canadian media showed similar recent trends to increasingly report on the need for rule changes and the need to protect youth ice hockey players from TBI.

## Discussion

We found several important trends about the reporting of TBIs in ice hockey. There has been a shift in not only reporting brain injuries when they occur to star players but also in reporting them more broadly across a variety of levels of skill. There is also a trend to recognizing the long-term severity and impact of TBI to the player and the need to take action against aggression, particularly at the youth levels. However, at the same time, there is a persistence of the theme that head injuries are *just a part of the game*, and that anyone who plays ought to just accept this occupational risk or not play. Exposure to these conflicting views may make it difficult for the reader to adopt a stance on the issue of sport-related TBIs. This can be concerning as it has been shown that media messages that create confusion in the population can lead to unhealthy behaviours [30]. Furthermore, a shift in focus on equipment as a protective device to a potential cause of more aggressive behavior was seen in both countries. A shift in concern towards making youth play safer through rule changes was manifest recently in both countries and a call on the professional league to take responsibility and action towards effective solutions was also seen in the recent newspaper articles. The articles that we analyzed echo the recent shift in scientific literature that documents the detrimental effects of TBI in sport [31], [32], [33]. The effect of TBI on the cognitive and psychosocial development of *youth* also seems to be accurately portrayed in the analyzed newspaper articles [5], [6]. Reports of brain injury as an unchanging “occupational hazard” reflect a stark contrast to the calls for rule changes and action by the most elite professionals in these newspaper articles.

Several useful models help us to understand how the media shapes individuals' knowledge, attitudes, and practices of injury-related risk-taking. Iyengar's (1991) model focuses on the intended and real effects of media including information-provision, setting agendas, framing, and persuasion [34]. By contrast, McGuire's (2001) approach considers the following factors important to media impact: source (credibility), message and content, channel, and audience variables [35]. Together, these models describe two dimensions of communication relevant to understanding the impact of the media on individuals' knowledge, attitudes, and practices of risk-taking: the intended and real effects (at an intermediate or macro-social level) of communication, as well as the qualities of presentation, content, and context that have been shown to produce changes in individuals' opinions and behaviours.

We are sensitive to Herman and Chomsky's classic argument that media discourse can be biased and reflect the interests of power elites including government officials and corporate or industry groups [36]. We like others, found that media reports often emphasized the aggressive and violent nature of games often in what seemed to be means to incite interest in the event by as many people as possible.

The role that such media reports have on youth attitudes and the culture of hockey cannot be ignored. While we recognize that family and close personal friends can influence individuals' formation of opinions and judgments, we also share Katz and Lazarfield's (1955) argument that individuals' interpretation of media messages can be directly shaped by opinion leaders in their communities as reported in the media [37]. Since young media consumers are particularly impressionable [38], it is not surprising that research has shown that youth exposed to themes of aggression and violence in the media are more likely to develop tendencies of physical aggressiveness, violent and delinquent behaviour, and conduct issues [39]. Furthermore, studies show that youth who are repeatedly exposed to violence and aggression in the media view violence and aggression as the appropriate means for solving conflict in all aspects of life [40]. So, it is likely that the reporting on TBI that we have documented is also a likely factor that contributes to a culture that normalizes aggressive and violent behaviour [41], [42].

Media reporting on health issues can also help shape positive health-related attitudes and behaviours [43]. During the SARS crisis, media reports on this disease outbreak were largely responsible for altered consumer behaviours, causing many to change their travel plans to avoid areas that the media decreed highly infectious [44]. Chapman et al. (2005) found that in four Australian states, in the two weeks after the media announced singer and pop-icon Kylie Minogue was diagnosed with breast cancer, bookings for mammographies increased by 40% [45]. This sort of reporting may also have unhealthy effects [46]. Jordan et al. (2008) propose that the media has contributed to the childhood obesity pandemic by advertising the unhealthy foods and beverages that children now demand and regularly consume [47]. This suggests that media reports of an issue such as TBI in sport can contribute to an altered culture. Those charged with promoting healthy behaviors would benefit from understanding these trends in the media reports.

The results and implications of this study need to be considered in light of the investigation's limitations. Although the newspapers analyzed in this investigation are highly circulated, they represent only a small proportion of all daily, paid North American newspapers. Additionally, we only reviewed the articles of 4 largely circulated newspapers – it is not clear whether similar trends were reported in other newspapers, on radio, television and through other online sources. To assess the impact on such media reports on the public would require prospective studies with large numbers of people, a study beyond the scope of the present investigation.

## Conclusion

We have shown that reporting of TBI and its context has changed substantially over time. That our findings were consistent within and across countries, with scientific reporting around TBI and over time provides evidence for the robustness of our findings. Future work that builds upon our findings should focus on how reporting of TBI in ice hockey can affect public discourse and the shaping of programs and policies that have positive effects on public health.

## Dedication

This work is dedicated to the memory of Randy Starkman, the Toronto Star's veteran athletics journalist and a two-time National Newspaper Award winner, who died April 16, 2012.
